# Notes on syphilis vaccine development

**DOI:** 10.3389/fimmu.2022.952284

**Published:** 2022-07-28

**Authors:** Noah Kojima, Kelika A. Konda, Jeffrey D. Klausner

**Affiliations:** ^1^ Department of Medicine, University of California Los Angeles, Los Angeles, CA, United States; ^2^ Centro de Investigación Interdisciplinaria en Sexualidad Sida y Sociedad, Universidad Peruana Cayetano Heredia, Lima, Peru; ^3^ Departments of Medicine and Population and Public Health Sciences, Keck School of Medicine of the University of Southern California, Los Angeles, CA, United States

**Keywords:** syphilis, *treponema pallidum*, vaccine, vaccines, *treponema pallidum* antibodies

## Abstract

The quest for a syphilis vaccine to provide protection from infection or disease began not long after the isolation of the first *Treponema pallidum* subspecies *pallidum* (*T. pallidum*) strain in 1912. Yet, a practical and effective vaccine formulation continues to elude scientists. Over the last few years, however, efforts toward developing a syphilis vaccine have increased thanks to an improved understanding of the repertoire of *T. pallidum* outer membrane proteins (OMPs), which are the most likely syphilis vaccine candidates. More has been also learned about the molecular mechanisms behind pathogen persistence and immune evasion. Published vaccine formulations based on a subset of the pathogen’s OMPs have conferred only partial protection upon challenge of immunized laboratory animals, primarily rabbits. Nonetheless, those experiments have improved our approach to the choice of immunization regimens, adjuvants, and vaccine target selection, although significant knowledge gaps remain. Herein, we provide a brief overview on current technologies and approaches employed in syphilis vaccinology, and possible future directions to develop a vaccine that could be pivotal to future syphilis control and elimination initiatives.

## Introduction

Syphilis, caused by the spirochete *Treponema pallidum* subspecies *pallidum* (*T. pallidum*), remains a significant global and public health problem. The disease is still endemic in low- and middle-income countries and resurgent in high-income ones. In the United States, the number of syphilis cases in 2020 was the highest since 2000 (1). Furthermore, congenital syphilis transmission is the most common infection associated with fetal loss or stillbirth in low-income settings, with an estimated 1.4 million pregnant women infected every year globally, resulting in an estimated 305,000 prenatal or perinatal deaths, and 215,000 infants born prematurely and/or with clinical signs of syphilis (2, 3). Past public health initiatives to eliminate syphilis and congenital syphilis promoted by the Centers for Disease Control and Prevention (CDC) and the World Health Organization (WHO), have significantly reduced syphilis incidence, but have not achieved their intended elimination goals. Thus, new tools are needed to aid the currently available diagnostic tests and therapeutic options.

The only successful study of syphilis vaccination was reported by Dr. James Miller in 1973 (4). In his study, Miller used an extensive immunization regimen, injecting rabbits intravenously 60 times over 37 weeks with γ-irradiated *T. pallidum* cells. Immunization was followed by intradermal homologous challenge with the same *T. pallidum* strain. Immunized rabbits displayed complete protection that persisted for at least one year, as shown by the lack of development of chancres at the challenge sites and the absence of clinical/serological evidence of syphilis.

## *T. pallidum* proteomic array to study humoral immunity to syphilis

Evidence that repeated exposure to *T. pallidum* is conductive to partially protective immunity was also provided by human inoculation experiments conducted by Magnuson *et al.* in the 1950’s (5), which showed that a previous syphilitic infection of sufficiently long duration would produce significant protection against subsequent challenge. Others have found that in patients with repeated episodes of syphilis, reinfections led to less severe skin manifestations than in patients at their first syphilis episode (6). The evidence that previous syphilis attenuates the clinical manifestations associated with reinfection has been recently reiterated by Marra et al. (7) The study of how the immunological response to *T. pallidum* antigens differs in patients diagnosed with active syphilis but with and without a history of the disease could provide a basis for vaccine development. By increasing the understanding and describing the differences in immune responses between those two groups, researchers hope to identify unique aspects of the adaptive humoral response to inform vaccine development. If this protective immunity could be replicated through vaccination, the result could be a vaccine that leads to attenuation of early symptoms and, hence, decreased infectivity and transmission of *T. pallidum* (8). Therefore, a vaccine that can only induce partial protection, but can prevent early symptoms, and possibly pathogen dissemination could result in reduced syphilis spread and be of substantial public health benefit for populations at increased risk of syphilis.

Among the newest tools to study syphilis immunology is a *T. pallidum* proteomic array developed by Antigen Discovery Inc. (ADI) in collaboration with the Giacani laboratory (9). That array is conceptually not dissimilar from that described by Brinkman et al. in the early 2000’s based on the Nichols strain genome. However, the ADI array covers 99% of the proteome of two strains of *T. pallidum* (Nichols and SS14) and is amenable to high throughput analyses with mere microliters of serum. The array is currently being evaluated with longitudinal serum samples from long-term infected rabbits, and longitudinal serum samples from rabbits that were infected, treated with benzathine penicillin G (BPG) weeks after infection, and then re-infected after residual antibiotic was cleared. Furthermore, the array is being used to evaluate reactivity to ~150 clinical serum samples provided by our group and collected in Peru. Comparison of the differential reactivity to *T. pallidum* antigens (with emphasis on putative vaccine candidates) in serum from patients with and without a history of syphilis at diagnosis will help pinpoint protective antigens to be tested in pre-clinical vaccination/challenge experiments. Additionally, the ADI system for cell-free synthesis of *T. pallidum* antigens could be adapted to the investigation of *T. pallidum* antigens inducing a robust cellular response during infection. Those antigens could be added to a vaccine formulation to promote T cell activation and IFN-γ production to activate macrophages.

The usefulness of an instrument like the ADI proteomic array becomes far more remarkable when a vast array of well-characterized clinical samples is available. The institution of centralized biospecimen repositories with serum and lesion swab specimens would greatly benefit research efforts to understand syphilis immunology and genetic diversity in this pathogen.

## Genetic diversity and vaccine development

The publication of the Nichols strain genome sequence in 1998 by Fraser et al. (10), opened the gateway to reverse syphilis vaccinology. The conserved region of the variable antigen TprK (encoded by the *tp0897* gene) was one of the first vaccine candidates identified with that approach and it is still included in several modern experimental vaccine formulations (11, 12). Some preliminary work has been conducted to improve expression of surface-exposed integral outer membrane proteins like TprK and Tp0435 genes on engineered non-infectious *Borrelia burgdorferi* strains (13). Partial protection was observed in rabbit models that underwent immunization with *B. burgdorferi* that expressed TprK, while those that were immunized with Tp0435 did not have the same protection. Analysis of the humoral response to TprK antigen suggested there was reactivity to conformational epitopes to the antigen.

Dr. Caroline Cameron pioneered the application of genome-wide-bioinformatics analyses to predict *T. pallidum* open reading frames (ORFs) encoding putative outer membrane proteins (OMPs) to identify adhesins that would interact with receptors among the host extracellular membrane components (14). The result of that study was the identification of the Tp0751 adhesin. Lithgow et al. showed that Tp0751-immunized animals had a significantly reduced *T. pallidum* organ burden upon infectious challenge compared with unimmunized animals (15). Although not unanimously accepted by the research community (16), Tp0751 remains a current vaccine candidate worthy of further exploration. Additional *in silico* OMP-mining work further contributed to defining the putative repertoire of *T. pallidum* surface-exposed integral membrane proteins (17), which today consists of 27 proteins—the current best candidates for syphilis vaccine development, which are made up of components of the BAM complex, Lpt complex, 9-stranded beta-barrels, FadL-like protein, components of the efflux systems, and *T. pallidum* repeat proteins (18).

As complete and partial genomes from historical and modern isolates accumulated over the last two decades, it became increasingly clear that substantial genomic diversity was concentrated within the genes of *T. pallidum* encoding for surface-exposed proteins (19–21). This evidence has profound implications for developing a broadly protective vaccine and emphasizes the necessity to obtain high-quality, near-complete genomes that can be used to assess OMP variability. The sequence of several *T. pallidum* OMP-encoding genes has been notoriously challenging to elucidate through whole genome sequencing due to the presence of repetitive sequences. Several ongoing initiatives are addressing the necessity to obtain more *T. pallidum* genomes to refine vaccine development. Upon completing those efforts, the vaccine research community will benefit from a vast array of genomes, primarily obtained directly from patient samples and from many diverse geographical areas spanning all continents, including areas where syphilis is endemic.

Modern approaches that combine *T. pallidum* DNA enrichment with pathogen-specific probes (19, 22), specific genome amplification before high-throughput sequencing and technologies capable of sequencing Kb-long DNA molecules will ensure the availability of complete high-quality genomes for comparative genomics analyses (23–28). Deposition of reads and assembled genomes in public data repositories will enable more researchers to participate in vaccine development. A syphilis vaccine will likely need to be tailored to the genetic pedigree of strains circulating locally. On the upside, *T. pallidum* modern strains continue to share over 99% of genomic identity if we exclude the hypervariable gene *tprK*, which undergoes intra-strain gene conversion to foster *T. pallidum* persistence (29–31). Evidence supports two major clades of this pathogen circulating worldwide, the SS14-like and Nichols-like clade (28). The recent strain sequencing work by Lieberman *et al.* with *T. pallidum* isolates from Peru, Ireland, USA, Papua New Guinea, Madagascar, Italy, Japan, and China were included in the 196 near-complete genomes sequenced from eight countries and six continents (19).

## Omics and other approaches to the rescue

As the genomics gap is being closed at unprecedented speed for *T. pallidum*, the application of other “-omics,” mainly transcriptomics and proteomics, will provide complementary information to help identify vaccine candidates (32, 33). Only limited work to date has focused on analyzing the *T. pallidum* transcriptome and proteome (32–38). Yet those studies are pivotal to understand the timing and level of expression of potential immunogens. In addition to being poorly expressed, selected OMP-encoding genes have been reported to undergo stochastic modulation of gene expression through phase variation, which might contribute to changing the pathogen surface antigenic profile (39, 40). To date, a microarray study describing transcriptome of Nichols strain was conducted and found that the RNA transcript of *T. pallidum* profiles between *in vitro* culture and rabbit infection were similar (33). A better understanding of gene regulation and gene expression could lead to exclusion of specific vaccine candidates whose transcription might be turned off with no detriment to the pathogen.

To date, transcriptional profiles of *T. pallidum* have been obtained from rabbit or *in vitro*-propagated strains (33). Although those studies are valuable in improving our understanding of gene expression in *T. pallidum*, an equally important endeavor would be to assess gene expression in spirochetes from patient samples. Transcriptomics of clinical samples can be challenging: the small amount of clinical material obtained from clinical samples often precludes sequencing of the entire genome. Producing high quality whole genome sequencing data needs advanced molecular techniques for selected whole genome amplifications and bait enrichment of libraries to enable gathering meaningful data from clinical samples. At the same time, determination of levels of paralogous genes will be also very challenging as many tpr genes share identical sequences and some undergo recombinations.

An analysis of the *T. pallidum* transcriptome using bacterial cells present in lesions from individuals diagnosed with early syphilis, without strain propagation in rabbits, could also help better understand the immunology of natural infection. That work would inform whether specific gene expression patterns correlate to the development of the immune response and disease manifestations during early syphilis and, more generally, which vaccine candidates are expressed during different stages of the infection.

Successful genetic engineering of *T. pallidum* was reported in 2021 (41). Genetic manipulation of *T. pallidum* has the potential to pinpoint vaccine candidates. Efforts have been made to ablate the *tprK* ORF with no avail, which led to the hypothesis that *TprK* is an essential *T. pallidum* gene. That hypothesis is also supported by the evidence that extensive *tprK* sequencing never yielded a variant carrying an early termination due to a premature stop codon or a frameshift mutation, despite the extensive recombination events that involve this hypervariable gene. A vaccine design based on an OMP necessary for pathogen viability could be preferred to a design based on a non-essential gene. Current experiments to assess the “essential” OMP repertoire are ongoing in the Giacani laboratory through genetic engineering. New molecular tools for *T. pallidum*, such as transposon-mediated insertional mutagenesis, GFP-expressing *T. pallidum* cells, and a *T. pallidum* strain expressing constitutively spell out Cas9, are also being evaluated to accelerate the discovery of genes that, albeit not essential, might be necessary for *T. pallidum* virulence.

## Protein structure and vaccine development strategies

Immunization with recombinant treponemal proteins would greatly benefit from increased knowledge of the native structure of the candidate immunogens. However, no conclusive experimental data exist on the structure of *T. pallidum* OMPs. For about half of these molecules, the level of homology with other bacterial proteins has been sufficient to obtain high-confidence models using a battery of mainstream computational and bioinformatic tools (18, 42). On the contrary, there is an ongoing debate concerning the structure of Tpr antigens because the structure that is inferred from functional assays differs from that hypothesized based on structural data from protein fragments. Refining the structural models for all *T. pallidum* OMPs is therefore pivotal for vaccine development. High-confidence models will allow the excision of surface-exposed epitopes to be mounted on a carrier that is easier to produce than a recombinant OMP, contains fewer amino acid sequences that are not instrumental to developing a protective response, but maintain the structural characteristics of the native epitopes to allow the development of antibodies to conformational epitopes. Carriers such as viral-like particles, small beta-barrel antigens, liposomes, and outer membrane vesicles are all options worth trying.

## Adjuvants

Which adjuvant to use in a syphilis vaccine is also an issue that requires additional experimentation. Currently as vaccine research focuses on rabbit models, experimentation is conducted using ribi, titermax, or SAS, none of which are approved for human use. Experimentation has made clear that an adjuvant necessary to induce a Th1 response that will lead to INF-γ activated macrophages is crucial to an effective vaccine (43–46). Any vaccine formulation that includes adjuvants not suitable for humans will eventually have to be retested with adjuvants approved for human use. The reliance on a rabbit model for vaccine discovery leads to inevitable but necessary gaps in the potential for vaccine formulations to advance to human trials.

## Vaccine efficacy and target populations

To provide a favorable risk to benefit ratio, vaccines need to be safe for users and effective in preventing disease. For other vaccinations, a threshold of 50% efficacy rate had been determined to be adequate by large governing bodies (47). Given the need for syphilis vaccines in a global setting, heat-stable vaccines would greatly benefit distribution.

Furthermore, syphilis epidemiology is different in high-income versus low- and middle-income countries. In high-income countries, syphilis predominantly affects men who have sex with men (MSM), while in low- and middle-income countries, where the disease is endemic, syphilis impacts the general population. In implementing a syphilis vaccine, especially one that is only partly effective, it would be sensible to have different distribution strategies between high-income and low- and middle-income countries. In high-income countries, immunization should target those at increased risk for syphilis such as MSM and sex workers. In low- and middle-income countries, vaccination of the general population with a focus on protecting those of reproductive age to decrease risk of congenital syphilis would be recommended. With recent increases in congenital syphilis in the United States, vaccination of women of reproductive age may also be worthwhile.

## Cost analysis/Mathematical modeling

A mathematical model was created for a hypothetical syphilis vaccine assuming an efficacy of 80%. That study focused on vaccination in Africa, using different estimates of the prevalence of HIV infection in the general population (1.5%, 10%, and 15%). Syphilis vaccination reduced syphilis incidence for all the studied scenarios. However, focusing solely on young women or only on high-risk populations, was not as impactful on syphilis prevalence over time as mass vaccination (48). Additional work is needed to understand better how vaccines with differing efficacy and/or the reduction of clinical symptoms of syphilis can reduce transmission.

## Concluding remarks

Despite the low costs associated with syphilis testing and treatment, syphilis control has remained elusive. The years of life lost due to congenital syphilis are substantial (49). A vaccine able to reduce syphilis incidence, especially congenital syphilis, could significantly improve public health and lower the estimated 3.6 million disability-adjusted life years that are currently lost annually due to this serious infection.

## Author contributions

NK, KK, JK: writing and editing. All authors contributed to the article and approved the submitted version.

## Funding

This work was supported by US National Institutes of Health though the National Institutes of Health/National Institute of Allergy and Infectious Diseases, grant number 5R01AI139265.

## Acknowledgments

The authors are grateful to Dr. Lorenzo Giacani (University of Washington) for his suggestions to improve this manuscript.

## Conflict of interest

The authors declare that the research was conducted in the absence of any commercial or financial relationships that could be construed as a potential conflict of interest.

## Publisher’s note

All claims expressed in this article are solely those of the authors and do not necessarily represent those of their affiliated organizations, or those of the publisher, the editors and the reviewers. Any product that may be evaluated in this article, or claim that may be made by its manufacturer, is not guaranteed or endorsed by the publisher.
